# Reducing the airflow waveform distortions from breathing style and body position with improved calibration of respiratory effort belts

**DOI:** 10.1186/1475-925X-12-97

**Published:** 2013-09-28

**Authors:** Tiina M Seppänen, Olli-Pekka Alho, Tapio Seppänen

**Affiliations:** 1 Department of Computer Science and Engineering, University of Oulu, Oulu, Finland; 2 Department of Otolaryngology, University of Oulu, Oulu, Finland

**Keywords:** Calibration, Regression, Respiration, Spirometer, Flow, Rib cage, Chest, Abdomen

## Abstract

**Background:**

Respiratory effort belt measurement is a widely used method to monitor respiration. Signal waveforms of respiratory volume and flow may indicate pathological signs of several diseases and, thus, it would be highly desirable to predict them accurately. Calibrated effort belts are sufficiently accurate for estimating respiratory rate, but the respiratory volume and flow prediction accuracies degrade considerably with changes in the subject’s body position and breathing style.

**Methods:**

An improved calibration method of respiratory effort belts is presented in this paper. It is based on an optimally trained FIR (Finite Impulse Response) filter bank constructed as a MISO system (Multiple-Input Single-Output) between respiratory effort belt signals and the spirometer in order to reduce waveform errors. Ten healthy adult volunteers were recruited. Breathing was varied between the following styles: metronome-guided controlled breathing rate of 0.1 Hz, 0.15 Hz, 0.25 Hz and 0.33 Hz, and a free rate that was felt normal by each subject. Body position was varied between supine, sitting and standing. The proposed calibration method was tested against these variations and compared with the state-of-the-art methods from the literature.

**Results:**

Relative waveform error decreased 60-70% when predicting airflow under changing breathing styles. The coefficient of determination R^2^ varied between 0.88-0.95 and 0.65-0.79 with the proposed and the standard method, respectively. Standard deviation of respiratory volume error decreased even 80%. The proposed method outperformed other methods.

**Conclusions:**

Results show that not only the respiratory volume can be computed more precisely from the predicted airflow, but also the flow waveforms are very accurate with the proposed method. The method is robust to breathing style changes and body position changes improving greatly the accuracy of the calibration of respiratory effort belts over the standard method. The enhanced accuracy of the belt calibration offers interesting opportunities, e.g. in pulmonary and critical care medicine when objective measurements are required.

## Background

Respiratory effort belts are a convenient way to monitor respiration noninvasively, unobtrusively and continuously. This is especially important for examination of different kinds of respiratory disorders, like airway obstructions and impending respiratory failure in critical care patients. Respiratory effort belts are often used, for example, to monitor the respiration in sleep, in children and infants, and to detect the functional disorders of the respiratory system and the respiratory muscle dysfunctions [1-3].

Respiratory effort belts are elastic belts that are placed around the rib cage and abdomen of the subject to measure dimensional changes during breathing. After calibration to spirometer or pneumotachograph signal they can be used quantitatively to measure respiratory volume and flow [3,4]. The relation between breathing and belt dimensional changes can be determined by modeling the respiratory system as two degrees of freedom system with two moving parts: rib cage and abdomen [5]. The volumetric changes of the respiratory system can be predicted as the weighted sum of the dimensional changes of the rib cage and abdominal compartments. Consequently, the respiratory volume measured at the mouth is related to the sum of the volume changes of the rib cage and abdomen, while the derivative of the respiratory volume yields respiratory flow. This concept of two degrees forms the basis of various computational techniques that can be used to calibrate the respiratory effort belts, e.g. isovolume [6,7], least-squares [7,8], and multiple linear regression techniques [7,9-11].

It would be highly desirable to predict respiratory volume and flow signal waveforms accurately during tidal breathing as, because they contain pathological signs, for example, of asthma [12], airway obstruction [13-15], cystic fibrosis [16], and chronic obstructive pulmonary disease [17]. However, the respiratory volume and respiratory flow accuracy are degraded with subject body position or breathing style change due to the change of calibration factors [18]. Another element that may impair the prediction accuracy of the respiratory volume by respiratory effort belts is the thoracoabdominal asynchrony (TAA) that is often observed in many respiratory disorders and/or respiratory muscle dysfunctions. TAA refers to the non-coincident motion of the rib cage and the abdomen and is characterized by a time lag between motion of the rib cage and the abdomen. TAA is clinically assessed as a sign of respiratory distress and increased work of breathing [2]. The asynchrony disturbs the relationship between the thoracoabdominal movement and the respiratory volume, and thus, makes accurate calibration difficult [19].

Respiratory rate and tidal volume accuracy have been studied extensively, but the ability of the calibration methods to derive waveform of respiratory flow signal or volume signal has been studied much less. The exact waveform shape is a requirement for the estimation of the two most referenced tidal breathing parameters: the ratio of the time to peak tidal expiratory flow to the total expiratory time (*t*_*PTEF*_*/t*_*E*_) and the ratio of the respiratory volume at the peak tidal expiratory flow to the expired tidal volume (*V*_*PTEF*_*/V*_*E*_) [20]. Noninvasive respiratory volume signal waveform has been compared with the pneumotachograph flow signal using respiratory inductive plethysmograph in a few published articles [11,21,22].

The multiple linear regression technique with two predictor variables has been used commonly since the 1980’s for calibration of respiratory effort belts. However, this method yields only crude predictions of the waveforms in the airflow signal. If the breathing style changes from the calibration breathing style, predicted airflows get even worse [18]. To improve the accuracy of airflow prediction, efforts to modify the original two degree of freedom model have been made by adding parameters to the regression model. These parameters include for instance the parameter relating lung volume changes from the start of the inspiration or expiration to the rib cage and abdominal excursions from initiation of respiratory motion [11]; an interaction term between the rib cage and abdominal compartments [23]; and eleven features directly or indirectly related to ventilation such as volume, frequency and body size features [24]. However, challenges with the accuracy still remain.

Here, we present an improved calibration method of respiratory effort belts which is an extension to the multiple linear regression method with two predictor variables: rib cage and abdominal respiratory effort belt signals. The method is based purely on the belt signals and does not use any other information source for calibration. In this study, we concentrate on challenging situations where the breathing style or body position changes. We assessed changing breathing styles, including metronome guided breathing, free breathing in different body positions, free breathing in dynamically changing body positions, and thoracoabdominal asynchrony with metronome guided breathing. The proposed method was compared with two reference methods: the standard multiple linear regression method and with a recent method of Liu *et al.*[24]. The performance was assessed, firstly, using a subject-specific approach, where the method was trained and tested for each subject separately, and secondly, using a subject-independent approach in which all data, expect that of a test subject, were used for model training.

## Methods

### Proposed calibration method

The relationship between the respiratory airflow from spirometer and the dimensional changes of the respiratory effort belt signals is conventionally modeled by applying the method of multiple linear regression [7]. The relationship is established by fitting the following model to the time-synchronized signals:

(1) $$y\;=\;\beta_{1}x_{1}+\;\beta_{2}x_{2}+\;\epsilon \;=\;x^{T}\beta \;+\;\epsilon$$

where *y* denotes respiratory airflow from spirometer, the respiratory effort belt signals *x*_1_ and *x*_2_ from the rib cage and abdomen, respectively, are the predictor variables, β_i_:s are regression coefficients and ϵ is a zero-mean Gaussian error. Superscript T denotes matrix transpose in the formula. In this standard model, one sample of each predictor variable is used at a time to predict the response variable.

**Figure 1:**
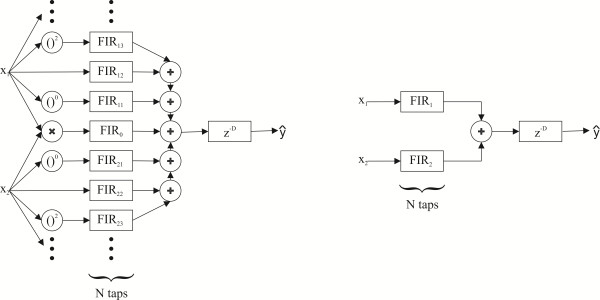
**Proposed method depicted as a MISO system.** (Left) Polynomial FIR filter bank for modeling the transfer function between respiratory effort belt and spirometer signals. (Right) A simplified structure for piezo-based and inductive-based respiratory effort belts. Here, FIR_1_ and FIR_2_ represent the filters FIR_12_ and FIR_22_ on the left, respectively.

Our proposed method is based on the MISO (Multiple-Input Single-Output) system model consisting of a polynomial FIR (Finite Impulse Response) filter bank and a delay element, see on the left in Figure 1. The proposed model extends the standard one in two important ways: (1) it uses a number N of consecutive signal samples and linear filtering for each prediction and (2) it is based on polynomial regression to model different transfer functions between the input and output. In the model representation, vector notation (bold letter type) is used below to denote that N consecutive signal samples of each predictor variable are included as components, and that the parameters are now vectors of dimension N. The model can be established as follows:

(2) $$y=\;f\left(x,\beta \right)+\epsilon$$

where *f* denotes the non-linear transfer function between the respiratory effort belts and spirometer. The transfer function is realized by the polynomial filter bank.

The rationale behind using the FIR filtering and polynomial regression is as follows: The standard prediction method essentially makes the assumption that the signal waveforms from the respiratory effort belts and spirometer are the same and takes a weighted average of the two inputs in order to make a prediction. However, the respiratory effort belts measure the thoracoabdominal movements and the spirometer measures airflow, and they use different kinds of sensor technique. For this reason, their signal waveforms are different. In addition, there are different kinds of commercial respiratory effort belt sensors such as piezo-based and inductive-based which produce further unpredictability to the accuracy. The FIR filters perform optimal linear transforms of the belt signal waveforms in order to match them to the spirometer signal waveform, which makes the result of the prediction much more accurate than with the standard method. In addition, with such sensors where the linear transform is not sufficient to correct the waveforms, selected nonlinear components can be included in the system, as depicted on the left in Figure 1. Another source of nonlinearity is the relationship between the cross-sectional areas of the effort belts and the actual lung volume. These additional filters input, e.g. intercept term, higher order polynomial terms and cross-product terms of the thoracoabdominal signals.

We recently observed that the addition of extra terms in the standard regression model did not improve results further when using our piezo-based or inductive-based respiratory effort belts [25]. This confirmed results published earlier [11,23]. In addition, these extra terms did not improve performance of our proposed method either [25]. Thus, the following model provides sufficient performance with our belts:

(3) $$y=\beta_{1}^{T}x_{1}+\beta_{2}^{T}x_{2}+\epsilon$$

Where $\beta_{1}^{T}$ and $\beta_{2}^{T}$ denote the N tap coefficients of filters FIR_1_ and FIR_2_ on the right in Figure 1, respectively. ***x***_1_ and ***x***_2_ are vectors including N consecutive samples from the rib cage signal and abdomen signal, respectively: ***x***_1_ = [*x*_11_, *x*_12_, …, *x*_1*N*_]^*T*^ and ***x***_2_ = [*x*_21_, *x*_22_, …, *x*_2*N*_]^*T*^.

The spirometer signal and the respiratory effort belt signals are input to the regression analysis which yields optimal tap coefficients and minimal prediction error for both filters. During the calibration, tap coefficients $\beta_{1}^{T}$ and $\beta_{2}^{T}$ are estimated with the method of least-squares from the available data. The least-squares estimator of ***β*** is given by

(4) $$\hat{\beta }\;={\left(X^{T}X\right)}^{-1}X^{T}y$$

where **y** is an n × 1 vector of observations from the spirometer signal, ***β*** is a (2  ×  N)  ×  1 vector of the tap coefficients and ***X*** is an n  ×  (2  ×  N) matrix of observations from the respiratory effort belt signals. Variable n is the amount of observations used to the calibration (in this study 3000 samples with 1 minute signal and sampling frequency 50 Hz). The parameter vector ***β*** is established as $\beta ={\left[\beta_{1}^{T},\beta_{2}^{T}\right]}^{T}$. Matrix ***X*** is formed as follows:

(5) $$X=\left[\begin{array}{cccccccc} x_{11} & x_{12} & \cdots & x_{1N} & x_{21} & x_{22} & \cdots & x_{2N}\\ x_{12} & x_{13} & \cdots & x_{1\left(N+1\right)} & x_{22} & x_{23} & \cdots & x_{2\left(N+1\right)}\\ \vdots & \vdots & \vdots & \vdots & \vdots & \vdots & \vdots & \vdots \\ x_{1n} & x_{1\left(n+1\right)} & \cdots & x_{1\left(n+N-1\right)} & x_{2n} & x_{2\left(n+1\right)} & \cdots & x_{2\left(n+N-1\right)} \end{array}\right]$$

The vector of predicted airflow ***ŷ*** is now given by

(6) $$\hat{y}\;=X\hat{\beta }$$

which is, thus, the spirometer signal predicted from the respiratory effort belt signals through the FIR filter bank.

There is a small delay between the respiratory effort belts signals and spirometer signal due to two reasons. One reason for the delay is that the spirometer signal is measured from the mouth and respiratory effort belt signals are measured from the rib cage and abdomen. Another reason is that there is the internal delay in each measuring device. For these reasons, the delay element z^-*D*^ is included at the output, see Figure 1. The delay was estimated by sliding the spirometer signal in relation to the respiratory effort belt signals within predetermined time limits sample by sample and at each step solving the regression problem. The minimum error *ϵ* in the respiratory flow prediction determined the optimal delay value.

In our previous study, the 0.3 sec time window of the FIR filters was found to produce the best respiratory airflow prediction when stable measurement data were used [25]. Thus, we used the same window size for the FIR filters also in this study. In addition, we tested the window size of 0.15 sec to find out whether the reduction of the trained parameters (tap coefficients) helps avoiding possible overtraining when the regression model is established with one breathing style and tested with others. With the used sampling frequency of 50 Hz, the number of tap coefficients was 16 and 8, respectively.

### Measurement devices

For calibrating the respiratory effort belt signals, the respiratory airflow signal was recorded with a spirometer (SpiroStar USB M9460, Medikro Oy, Kuopio, Finland). The sampling rate of the spirometer was 100 Hz. Respiratory effort belt signals were recorded with the Embletta Gold recorder (Embletta Gold, Denver, Colorado, USA), which had inductive respiratory effort belts for rib cage and abdomen and sampling rate of 50 Hz. All the signals were resampled at a common rate. The sampling rate of the Embletta Gold recorder was the lower one, 50 Hz, and was chosen as the basis rate. Consequently, the spirometer signal was decimated from 100 Hz to 50 Hz.

### Subjects and measurements

In planning and performing the study, the ethical principles regarding human experimentation were followed according to the Declaration of Helsinki. All the patients provided written informed consent and the study protocol was approved by the Oulu University Hospital ethics committee (reference number 83/2011). Ten healthy non-smoker adult volunteers (four females and six males) were recruited. The mean (SD) age of the subjects was 32 (4) years and they all were free of medications and had no heart or lung diseases. Volunteers gave a written informed consent and, additionally, brief background information was gathered using a questionnaire. Before the actual measurement session, subjects were not allowed to have caffeine products (coffee, tea, energy drinks) for the preceding 12 hours, alcohol for the preceding 24 hours, nor heavy meal and sport in the measurement morning.

**Figure 2:**
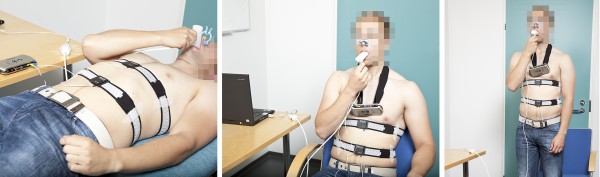
**Measurement setup with spirometer and respiratory effort belts.** Body positions from left to right: supine, sitting and standing.

All measurements were done between 8 and 12 A.M. Rib cage belt was placed on the xyphoid process and the abdominal belt was placed near the umbilicus. After that, subjects had a resting period of fifteen minutes in supine position before the actual measurements. During the measurement, respiratory effort belt signals were recorded along with the spirometer signal. The measurement protocol consisted of thirteen steps (Table 1). Breathing was varied between the following styles: metronome-guided controlled breathing rate of 0.1 Hz, 0.15 Hz, 0.25 Hz and 0.33 Hz, and a free rate that was felt normal by each subject. Body position was varied between supine, sitting and standing (Figure 2). The length of each measurement step was one minute during which the subject was instructed to stay put and not to move their limbs. There was always a 2–3 minute resting period between the steps. In this study, the protocol steps 1–12 were used. We made cross testing between different steps using the measurement data of one step to train the prediction model and that of some other step to test the prediction model. We made tests using a subject-specific approach and also in some cases a subject-independent approach. In the latter approach, one subject’s data is left out at a time, the data of all other subjects is used for training of the model, and the left-out observation is used for testing. This is also called leave-one-out validation.

**Table 1:** Measurement protocol

**Step**	**Body position**	**Breathing style**	**Frequency [Hz]**
0	Stabilization – supine	Free	
1	Supine	Free	
2	Supine	Free	
3	Sitting	Free	
4	Standing	Free	
5	Sitting	Free	
6	Sitting	Controlled	0.25
7	Sitting	Controlled	0.15
8	Sitting	Controlled	0.33
9	Sitting	Controlled	0.10
10	Sitting	Free	
11	Active standing up (30 s sitting, 30 s standing)	Free	
12	Active standing up (20 s sitting, 20 s standing, 20 s sitting)	Free	
13	Sitting	Free	

### Statistics

The spirometer signals and predicted airflows from respiratory effort belts were compared by computing R^2^ and RMSE (Root Mean Square Error) values. R^2^ is the coefficient of determination between the spirometer signal and the predicted airflow. The coefficient of determination is calculated from *R*^2^ = 1 - *SS*_*res*_/*SS*_*tot*_[26], where SS_res_ is the sum of squares of residuals between the spirometer signal and predicted airflow, and SS_tot_ is the total sum of squares calculated from the spirometer signal. RMSE is a measure of the difference between the spirometer signal and the predicted airflow. Relative RMSE [%] is the proportion of RMSE from RMS (Root Mean Square) of the spirometer signal. Furthermore, Bland-Altman plots were drawn to illustrate the difference between the proposed method and the standard one.

## Results and discussion

In the following subsections, the subject-specific approach is used unless otherwise stated in the subtitles. The proposed method was evaluated with different breathing styles and body positions. In accordance with that, the results were divided in the following subsections: (1) free breathing with unchanged body positions (sitting, supine); (2) metronome guided different breathing styles in sitting position; (3) subject-independent model with free breathing and metronome guided breathing in sitting position; (4) free breathing in different body positions (sitting, supine, standing); (5) free breathing with dynamic body position change (sitting, standing); (6) thoracoabdominal asynchrony of respiratory effort belts; and (7) subject-independent models with three different calibration methods.

### Unchanged body position

At first, prediction performance of free breathing in sitting and in supine position was assessed. In sitting position, data from step 5 was used to train the model and data from step 10 was used to test the model. In supine position, step 1 was used to train the model and step 2 to test the model. One subject’s rib cage belt signal contained so much of disturbances that we had to exclude that subject from supine position testing. Thus, there were data of ten subjects in sitting position testing and data of nine subjects in supine position testing. Table 2 summarizes the results from these tests. It is clearly seen in Table 2 that the proposed method (PM) with the FIR filter tap coefficients (N) 8 and 16 improved results greatly over the standard method (SM). In both body positions, R^2^ improved, relative RMSE decreased and relative respiratory volume error decreased. Additionally, standard deviation reduced in all cases indicating better predictability of the modeling error. Table 3 presents the relative improvements when predicted airflows produced with the different sizes of FIR filters were compared with the standard method. In both cases, R^2^ increased about 20% approaching 0.94 and relative RMSE decreased almost 50% when N values 8 and 16 were used. Additionally, the N value of 16 slightly increased R^2^ and decreased relative RMSE compared to the N value of 8.

**Table 2:** Results (average value ± SD) of the calibration with the standard method (SM) and proposed method (PM) with N values of 8 and 16 in unchanged body positions

**Body position**	**Method**	**R**^**2**^	**Relative RMSE [%]**	**Relative volume error [%]**
**Sitting**	SM	0.789 ± 0.050	45.7 ± 5.5	-9.2 ± 22.9
	PM(N=8)	0.935 ± 0.017	25.4 ± 3.4	-6.5 ± 10.4
	PM(N=16)	0.948 ± 0.011	22.7 ± 2.7	-4.1 ± 11.0
**Supine**	SM	0.792 ± 0.070	45.2 ± 7.0	1.8 ± 19.3
	PM(N=8)	0.937 ± 0.026	24.6 ± 5.3	-0.3 ± 7.3
	PM(N=16)	0.945 ± 0.027	22.7 ± 5.9	-0.4 ± 7.2

**Table 3:** Comparison of the standard method and proposed method in unchanged body positions

**Body position**	**Compared methods**	**ΔR**^**2**^	**Δ(relative RMSE)**
**Sitting**	SM→PM(N=8)	18.9 ± 7.7	-43.8 ± 9.1
	SM→PM(N=16)	20.6 ± 7.2	-50.0 ± 6.6
	PM(N=8)→PM(N=16)	1.4 ± 1.1	-10.4 ± 5.8
**Supine**	SM→PM(N=8)	19.1 ± 10.5	-45.3 ± 10.7
	SM→PM(N=16)	20.3 ± 10.6	-49.6 ± 11.8
	PM(N=8)→PM(N=16)	0.9 ± 0.5	-8.4 ± 5.3

The relative improvement of R^2^ and relative RMSE (average value ± SD, in [%]) between predicted airflows with the standard method and proposed method with N values of 8 and 16. The notation ´a→b´ denotes a performance improvement when changing from method ´a´ to method ´b´.

**Figure 3:**
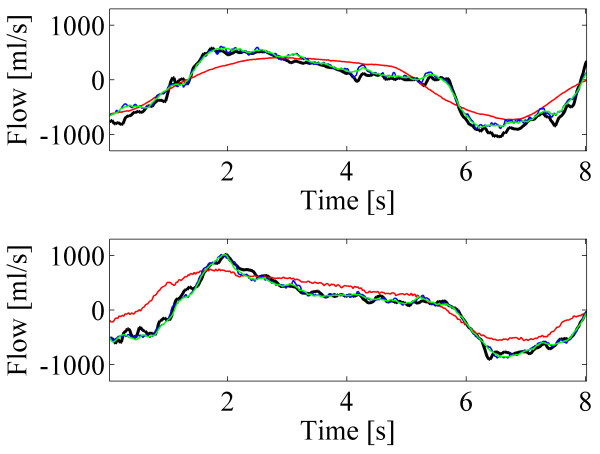
**Short segments of example signals in sitting (upper) and supine (lower) body positions.** Spirometer signals (black) and the predicted airflows (red: the standard method, blue: proposed method with N=8, green: proposed method with N=16).

Figure 3 depicts a short segment of example signals from both body positions. The predicted airflow with the N values 8 and 16 followed much more accurately the spirometer signal than that with the standard method. Predicted airflows with the N values of 8 and 16 are almost completely overlapping visually.

The results were consistent with our earlier findings indicating the proposed method improves the calibration accuracy greatly in the case of stable breathing and body position [25]. In the rest of the section we present results with the more challenging cases: changing breathing styles and body positions.

### Different breathing styles

To study the effect of different breathing styles, the protocol steps 5–10 were used. The model was trained with the data of each step at a time and tested with all the other steps containing different breathing styles. An average was taken over all combinations of the steps for each subject. Results from these tests are presented in Table 4. It is clearly seen that FIR filter size 8 and 16 improved results even more dramatically than in the stable body position cases above. However, the average of R^2^ values was negative for airflows predicted with the standard method, because in several of those cases, the residual was so large that R^2^ received negative values. Negative values may occur if the residuals between the spirometer signal and predicted airflow contain very large values (see section Statistics). This may happen when a regression model fails completely to predict data from very different body positions or breathing styles. When the predicted airflow was produced with the N values of 8 and 16, the R^2^ (average ± std) was 0.92 ± 0.024; and 0.93 ± 0.024, respectively. The relative RMSE decreased 61.5% when comparing the predicted airflow produced with the standard method to that of produced with the FIR filter size 8. Relative error of respiratory volume decreased from 17.1 ± 72.0% to -4.3 ± 13.1%, respectively. Corresponding values when comparing the predicted airflow produced with the standard method to that of produced with the N value of 16 were: the relative RMSE decreased by 63.8% and relative error of respiratory volume decreased from 17.1 ± 72.0% to -2.1 ± 13.1%. Thus, the standard deviation of respiratory volume error decreased 81% with proposed method indicating much better predictability of the respiratory volume. In addition, the relative RMSE decreased by 6.3% when comparing the predicted airflow produced with the N value of 8 to that of produced with the N value of 16.

**Table 4:** Results (average value ± SD) of the calibration with the standard method and proposed method with N values of 8 and 16 for each subject

**Subject**	**SM**		**PM(N = 8)**		**PM(N = 16)**	
	**Relative RMSE [%]**	**Relative volume error [%]**	**Relative RMSE [%]**	**Relative volume error [%]**	**Relative RMSE [%]**	**Relative volume error [%]**
1	78.6 ± 51.5	36.6 ± 81.4	29.2 ± 4.9	-5.6 ± 12.4	25.6 ± 4.7	1.2 ± 12.8
2	82.5 ± 50.1	20.7 ± 90.4	31.2 ± 11.4	-5.6 ± 7.2	29.5 ± 12.3	-4.5 ± 7.1
3	71.1 ± 37.7	25.4 ± 76.8	29.0 ± 4.0	-2.8 ± 11.8	26.3 ± 4.2	1.8 ± 14.1
4	73.0 ± 38.5	11.9 ± 74.1	29.7 ± 6.6	-3.6 ± 15.2	28.3 ± 5.5	-3.3 ± 14.5
5	73.0 ± 35.0	2.1 ± 64.0	26.6 ± 3.5	-5.0 ± 13.8	25.8 ± 4.4	-3.8 ± 13.7
6	64.6 ± 29.8	14.0 ± 66.2	21.7 ± 2.2	-2.2 ± 8.8	19.7 ± 1.9	-0.8 ± 8.7
7	70.6 ± 27.9	20.0 ± 66.5	22.2 ± 5.6	-0.4 ± 7.5	21.1 ± 4.8	1.1 ± 8.3
8	64.6 ± 33.1	11.9 ± 60.7	33.7 ± 10.9	-9.7 ± 25.9	32.6 ± 11.2	-8.1 ± 25.9
9	74.8 ± 43.0	16.0 ± 70.7	24.4 ± 6.1	-4.4 ± 13.2	22.7 ± 6.7	-0.1 ± 13.3
10	64.0 ± 32.9	12.0 ± 69.4	27.3 ± 4.0	-3.6 ± 15.3	26.2 ± 4.4	-4.7 ± 12.2
**Average**	71.6 ± 38.0	17.1 ± 72.0	27.5 ± 5.9	-4.3 ± 13.1	25.8 ± 6.0	-2.1 ± 13.1

The training and testing were performed using different breathing styles.

**Figure 4:**
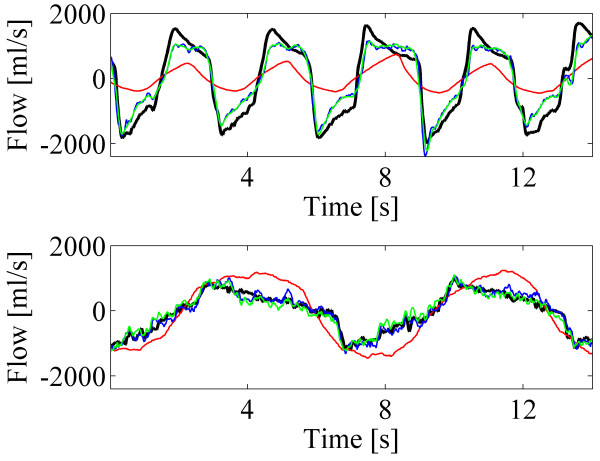
**Short segments of example signals from two different breathing styles.** Spirometer signals (black) and the predicted airflows (red: the standard method, blue: proposed method with N=8, green: proposed method with N=16).

Figure 4 depicts short segments of example signals from two subjects. The predicted airflows were computed with the standard method (red line), N value of 8 (blue line) and N value of 16 (green line). In the upper subfigure, the model was trained with the data of step 9 (breathing cycle 9.9 sec and respiratory volume 2.72 l) and tested with the data of step 8 (breathing cycle 3.0 sec and respiratory volume 1.58 l). In the lower subfigure, the model was trained with the data of step 8 (breathing cycle 3.0 sec and respiratory volume 1.73 l) and tested with the data of step 7 (breathing cycle 6.7 sec and respiratory volume 1.56 l). Predicted airflows with the N values of 8 and 16 clearly follow original spirometer signal much more accurately than that of with the standard method.

**Figure 5:**
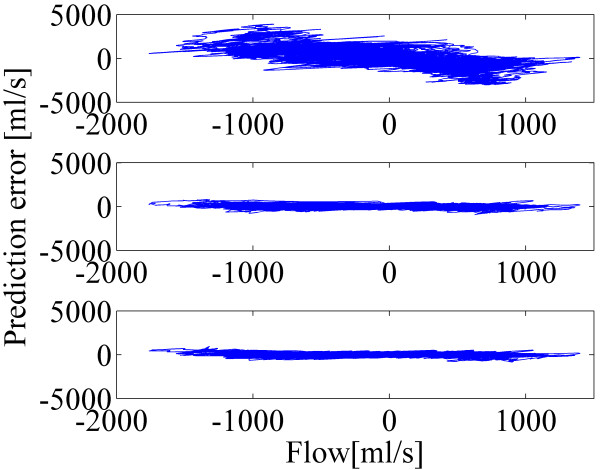
**Bland-Altman plots from calibration with the different breathing styles.** Spirometer signal is on the horizontal axis and the prediction error signal is on the vertical axis. Prediction error signals are computed for the predicted airflows with the standard method (top), proposed method with N=8 (middle) and proposed method with N=16 (bottom).

Bland-Altman plots in Figure 5 depict the effect of the proposed method with different size of FIR filters for the calibration. In all subfigures, the spirometer signal is on the horizontal axis and the prediction error signal (spirometer signal minus predicted airflow) is on the vertical axis. In this case, the training was done with step 8 (0.33 Hz) data and after that the predicted airflow was computed with the data from step 9 (0.1 Hz). The data was gathered from all ten subjects. By comparing the plots for the standard method and proposed method with N values 8 and 16, a clear distinction can be seen: the plots with the N values of 8 and 16 are much more compact and they have clearly less structure. This indicates the superiority of the calibration method proposed here.

### Subject-independent models

**Figure 6:**
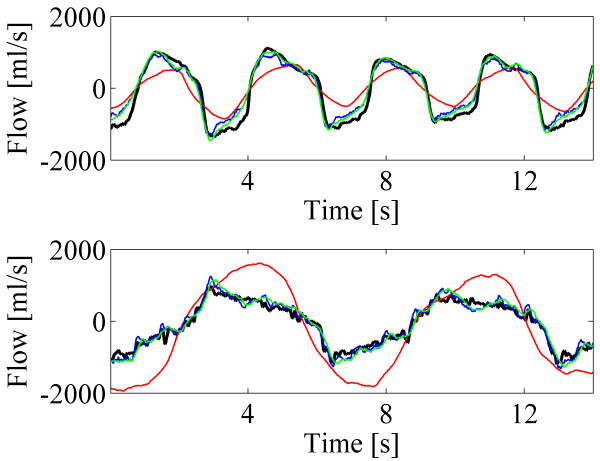
**Short segments of example signals from free breathing (upper) and controlled breathing (lower).** Spirometer signals (black) and the predicted airflows (red: the standard method, blue: proposed method with N=8, green: proposed method with N=16). Predicted airflows are computed with the subject-independent model.

Ten-fold cross-validation was applied in the subject-independent approach: data from measurements of nine subjects were used to train the prediction model and the testing of the model was done by using the data of one subject that was excluded from the training set. The average performance was finally calculated over all test subjects. Two tests were done: (1) free breathing with step 10 (training) and step 5 (testing); and (2) controlled breathing with step 8 (0.33 Hz breathing, training) and step 7 (0.15 Hz breathing, testing). An average was taken over all ten combinations in both tests. Results from these test cases are presented in Table 5. Proposed method with the N values 8 and 16 improve results greatly in both test cases. Especially the results from controlled breathing indicate radical improvements. In this case, the average of R^2^ values was negative for airflows predicted with the standard method, because in the most of those cases, error was so large that R^2^ received negative values. The relative RMSE decreased by 70.7% and 69.8% when comparing the predicted airflow produced with the standard method to that of produced with the N values of 8 and 16, respectively. Additionally, relative respiratory volume error and its standard deviation changed from 99.5 ± 51.3% (the standard method) to -0.2 ± 24.0% (N value of 8) and -1.2 ± 24.7% (N value of 16). Figure 6 depicts short segments of example signals. Calibration with the standard method produced much worse predicted airflow than with the proposed method.

**Table 5:** Results (average value ± SD) of the calibration with the standard method and proposed method with N values of 8 and 16 using the subject-independent model

**Breathing**	**Method**	**R**^**2**^	**Relative RMSE [%]**	**Relative volume error [%]**
**Free**	SM	0.694 ± 0.152	53.8 ± 13.5	14.7 ± 40.8
	PM(N=8)	0.890 ± 0.065	31.8 ± 10.0	-6.5 ± 21.9
	PM(N=16)	0.900 ± 0.056	30.3 ± 9.2	-0.3 ± 23.2
**Controlled**	SM	-0.259 ± 0.768	107.2 ± 35.1	99.5 ± 51.3
	PM(N=8)	0.894 ± 0.061	31.4 ± 9.2	-0.2 ± 24.0
	PM(N=16)	0.889 ± 0.055	32.4 ± 8.2	-1.2 ± 24.7

**Figure 7:**
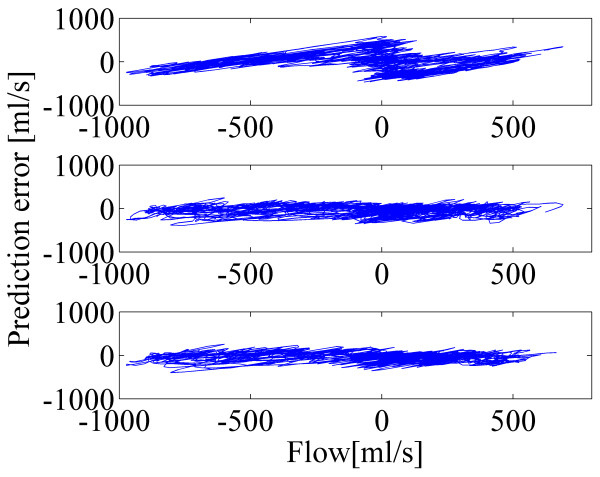
**Bland-Altman plots from calibration with the free breathing style and with subject-independent model.** Spirometer signal is on the horizontal axis and the prediction error signal is on the vertical axis. Prediction error signals are computed for the predicted airflows with the standard method (top), proposed method with N=8 (middle) and proposed method with N=16 (bottom).

**Figure 8:**
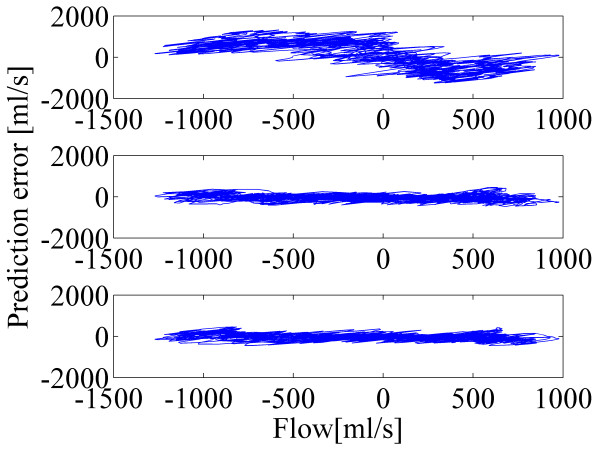
**Bland-Altman plots from calibration with the controlled breathing and with subject-independent model.** Spirometer signal is on the horizontal axis and the prediction error signal is on the vertical axis. Prediction error signals are computed for the predicted airflows with the standard method (top), proposed method with N=8 (middle) and proposed method with N=16 (bottom).

Bland-Altman plots in Figure 7 and Figure 8 depict the effect of the proposed method for the calibration. In both cases, by comparing the plots for the standard method, N values of 8 and 16, the superiority of the proposed method is clear: the plots with the N value of 8 and that of 16 are more compact and they have less structure. Distinction is even more evident when controlled breathing is used.

### Different body positions

The effect of different body positions was tested by using the supine (step 2), sitting (step 3) and standing (step 4) positions. The prediction model was trained with the data of one step and it was tested with the other two steps. An average was taken over all three combinations and ten test subjects. Results showing clear improvements are presented in Table 6. The R^2^ increased, relative RMSE decreased and the relative respiratory volume error decreased when values 8 and 16 for N was used. Additionally, standard deviations decreased in every case.

**Table 6:** Results (average value ± SD) of the calibration with the standard method and proposed method with N values of 8 and 16 in different body positions

**Method**	**R**^**2**^	**Relative RMSE [%]**	**Relative volume error [%]**
SM	0.654 ± 0.057	58.4 ± 15.4	-10.0 ± 15.9
PM(N=8)	0.876 ± 0.041	33.8 ± 8.3	-6.2 ± 5.6
PM(N=16)	0.885 ± 0.034	32.4 ± 9.6	-3.1 ± 7.4

### Changing body position

Data from step 11 and 12 in which the body position changed during the step, were used to test the prediction model trained with the data from step 10 (sitting). An average was taken over all results of ten subjects in both cases. Results from these cases are presented in Table 7. The relative RMSE values were now larger than in the previous cases but the values and their standard deviations were smaller with the N values 8 and 16.

**Table 7:** Results (average value ± SD) of the calibration with the standard method and proposed method with N values of 8 and 16 in body position change

**Step**	**Method**	**Relative RMSE [%]**	**Relative volume error [%]**
**11**	SM	88.0 ± 40.7	18.6 ± 40.5
	PM(N=8)	68.2 ± 20.7	-9.8 ± 9.4
	PM(N=16)	62.2 ± 19.1	-4.9 ± 7.2
**12**	SM	123.5 ± 47.1	83.4 ± 75.2
	PM(N=8)	92.5 ± 32.8	-5.3 ± 13.2
	PM(N=16)	86.6 ± 31.5	3.6 ± 12.3

**Figure 9:**
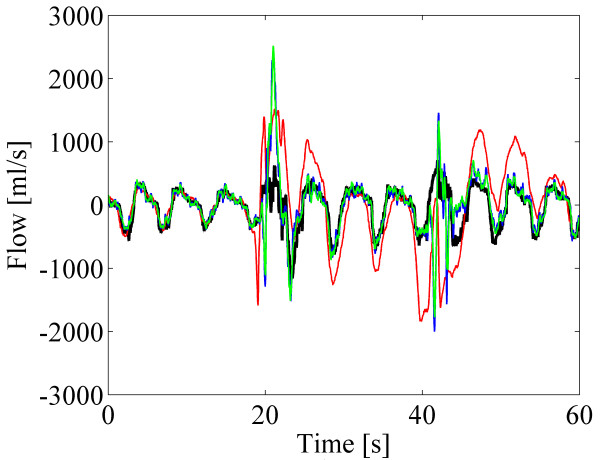
**Example signals from step 12 with body position changes.** Spirometer signal (black) and the predicted airflows (red: the standard method, blue: proposed method with N=8, green: proposed method with N=16).

Figure 9 depicts example signals from one subject from step 12. At the beginning when the subject was sitting, all predicted airflows follow the original spirometer signal quite well. All predicted airflows deviate from the spirometer signal at the time when body position changed (20 sec and 40 sec points in Figure 9) and proposed method with the FIR filter sizes 8 and 16 overestimate the flow for a short time period. However, the standard method overestimates the flow in a still longer time window. The predicted airflows produced with the proposed method then continue to follow the spirometer signal much more accurately than with the standard method.

### Thoracoabdominal asynchrony of respiratory effort belts

Thoracoabdominal asynchrony of respiratory effort belt signals was observed only in one subject’s measurement in step 9. In this step, the subject was breathing by metronome guidance in the speed of 0.10 Hz, which is considered unnaturally slow for most subjects. The training of the prediction model was performed with the data from the steps with synchronic belts signals: steps 5, 6, 7, 8 and 10. The testing of the model was performed with the data from step 9. An average was taken over all five combinations. Results from these cases are summarized in Table 8. Both relative RMSE and relative respiratory volume error decreased dramatically when proposed method with N values 8 and 16 were used. Yet, again the average of R^2^ values was negative for airflows predicted with the standard method. When N values of 8 and 16 were used, the R^2^ values were as high as over 0.9. The relative RMSE decreased by 81.6% and 83.1% when comparing the standard method and the proposed method with the N values of 8 and 16, respectively. Error in respiratory volume decreased from 154.4% (the standard method) to small fractions when N values 8 and 16 were used. Also, the standard deviations of relative RMSE and relative respiratory volume error decreased dramatically.

**Table 8:** Tests with asynchronous belt signals

**Method**	**R**^**2**^	**Relative RMSE [%]**	**Relative volume error [%]**
SM	-2.090 ± 2.241	165.8 ± 65.4	154.4 ± 83.2
PM(N=8)	0.906 ± 0.023	30.5 ± 3.6	-12.1 ± 4.9
PM(N=16)	0.920 ± 0.026	28.0 ± 4.4	0.8 ± 3.7

Results (average value ± SD) of the calibration with the standard method and proposed method with N values of 8 and 16.

### Comparison to reference methods with subject-independent model

The accuracy of the proposed method was compared with the one from Liu *et al*. [24] and the standard method of calibration [5]. Liu *et al*. have recently developed modified multiple linear regression models for estimating the minute ventilation of test subjects from data measured from rib cage and abdomen movements by sensor belts. They developed five models involving a combination of 11 features. Their results indicated that the inclusion of breathing frequency and the use of percentile points over 60 s data in the multiple linear regression model gave the most accurate results for their data. Thus the predictor variables used in our comparison are the best ones from their study: (1) the 10th percentile of an abdomen respiratory signal; (2) the 90th percentile of an abdomen respiratory signal; (3) the 10th percentile of a rib cage respiratory signal; (4) the 90th percentile of a rib cage respiratory signal; and (5) breathing frequency. Regression coefficients were estimated with the method of least-squares from the available data in all methods.

The subject-independent approach with the protocol steps 5–10 was used with one-subject-out cross-validation procedure. Data from nine subjects were used to train the prediction model and the testing of the model was done by using the data of one subject that was excluded from the training set. The regression coefficients were estimated with each of the method and the minute ventilation was predicted for each one-minute signal. The minute ventilation error [%] was then computed for all 60 cases from the predicted and measured (spirometer) minute ventilation. Results are presented in Table 9. It is clearly seen in the table that the average, standard deviation, median and MAD (Median Absolute Deviation) of minute ventilation error were the smallest when the proposed method was used. The standard method achieved a surprisingly small error on average but the variance of the error was large. A further advantage of the proposed method over the one from Liu *et al.* is that it can produce an accurate prediction of the breathing signal while the latter one is specialized in predicting the minute ventilation only.

**Table 9:** Comparison of the proposed method to the reference methods

**Method**	**Average**	**SD**	**Median**	**MAD**
Liu *et al*.	25.9	61.0	16.8	33.6
SM	3.6	74.8	-6.5	51.1
PM(N=8)	3.5	40.5	-3.4	25.9

Minute volume error [%] of the calibration with the method from Liu *et al*. [24], the standard method and proposed method (with N=8).

### Real-timeliness considerations

The methods were implemented in MATLAB® R2012b technical computing environment (MathWorks Inc., Natick, Massachusetts, U.S.A.). Average computing time (Intel® Core™2 Duo CPU P8600, 2.4 GHz) for the regression coefficients was 110 msec with the method from Liu *et al.* 120 msec with the standard method, 330 msec with the proposed method with filter size 8 and 880 msec with the proposed method with filter size 16.

Average computing time for the airflow prediction from one minute spirometer signal and respiratory effort belt signals was 0.2 msec with the standard method. Accordingly, average computing times for the proposed method with the filter size 8 and 16 were 1.1 msec and 1.7 msec, respectively. Average computing time for the prediction of minute ventilation was 0.01 msec with the method of Liu *et al*. 0.3 msec with the standard method and 1.1 msec with the proposed method with filter size 8. Computing time for all methods was only a fraction of a second for one minute measurement data which makes them suitable for real time applications.

## Conclusions

Here, we presented an improved calibration method that is an extension of multiple linear regression method. The method uses an optimally trained FIR filter bank and Multiple-Input Single-Output system model. The standard multiple linear regression method makes the assumption that the signal waveforms from the respiratory effort belts and spirometer are the same and takes a weighted average of the two inputs in order to make a prediction. However, the belts measure the thoracoabdominal movements and the spirometer measures airflow, and they use different kinds of sensor technique. For this reason, their output signal waveforms are different. In addition, there are different kinds of commercial belt sensors such as piezo-based and inductive-based which produce further unpredictability to the accuracy. Our FIR filters perform optimal linear transforms of the belt signal waveforms in order to match them to the spirometer signal waveform, which makes the result of the prediction much more accurate than with the standard method.

We demonstrated that the proposed method outperformed other compared methods with the prediction of minute ventilation. More importantly, the proposed method improves greatly the accuracy of the airflow prediction over the conventionally used one. The results showed that the improvement of the prediction accuracy is significant when the volunteers breathed freely in a stable body position. More importantly, when the different breathing styles were used, the prediction accuracy improved even more. In both cases, the predicted airflow computed with the proposed method followed much more accurately the original spirometer signal than the standard method. Consequently, not only the respiratory volume can be computed more precisely, but also the respiratory flow signal waveforms are very accurate. This offers an excellent opportunity to use respiratory effort belts for long term breathing measurements and produce more accurate waveform of respiratory volume and flow signals. This improvement may be particularly useful in the fields of pulmonary and critical care medicine.

## Abbreviations

FIR: Finite impulse response; MAD: Median absolute deviation; MISO: Multiple-input single-output; PM: Proposed method; RMS: Root mean square; RMSE: Root mean square error; SM: Standard method.

## Competing interests

The authors declare that they have no competing interests.

## Authors’ contributions

Development of method (TMS, OPA, TS); algorithm development (TMS, TS); computer programming (TMS); conception (TMS, OPA, TS); study design (TMS, OPA, TS); acquisition of data (TMS); analysis of data (TMS); interpretation of data (TMS, OPA, TS); drafting the manuscript (TMS); revising and finalizing the manuscript (TMS, OPA, TS). All authors read and approved the final manuscript.

## Authors’ information

TMS is MSc in Electrical Engineering. Currently, she works as a PhD student in the Biosignal Processing team of the Department of Computer Science and Engineering at the University of Oulu. OPA is a professor of otorhinolaryngology in the Department of Otolaryngology at the University of Oulu. TS is a professor of biomedical engineering in the Department of Computer Science and Engineering at the University of Oulu.
